# Work and Self-Rated Memory Among Native Hawaiian and Other Pacific Islander Older Adults

**DOI:** 10.1093/geroni/igaa057.1071

**Published:** 2020-12-16

**Authors:** Yeonjung (Jane) Lee, Tyran Terada

**Affiliations:** 1 University of Hawaiʻi at Mānoa, Honolulu, Hawaii, United States; 2 University of Hawai’i, Pearl City, Hawaii, United States

## Abstract

According to the productive aging framework, productive activities can function as protective factors of cognitive health. Productive activities, such as work, have been linked to positive cognitive health outcomes in older adults. Yet, less is known about if the beneficial effects of work on cognition extend to the Native Hawaiian and Other Pacific Islander (NHOPI) older adult population. Thus, the purpose of this research is to investigate how work is associated with self-reported memory/concentration among NHOPI older adults. Moreover, the moderating role of education was explored. Using data from the 2014 Native Hawaiian and Pacific Islander National Health Interview Survey (NHPI NHIS), the study explores the associations between work and self-rated levels of difficulty remembering or concentrating. A total of 1,045 older adults ages 50 years and older were included for analyses. Weighted multivariate analyses with multiple imputation techniques were used. The NHPI NHIS is the first federal survey focusing on the NHOPI population in the United States. Those who were engaged in work had lower odds of having severe difficulty memorizing or concentrating while controlling for other sociodemographic and behavioral factors. Interestingly, there was a significant interactive effect of work and education on self-rated memory. Those with lower education levels have lower self-rated memory, but the odds of having memory difficulty decreased when they worked. Findings highlight the importance of productive aging in promoting healthy cognitive aging. Research and practice addressing productive aging and cognition should provide culturally relevant resources to NHOPI older adults.

